# Supplementary material for: Impact of COVID-19 Vaccination on Short-Term Perceived Change in Physical Performance among Elite Athletes: An International Survey
Source: Vaccines (Basel). 2023 Apr 4;11(4):796. doi: 10.3390/vaccines11040796 (PMC10144569; doi:10.3390/vaccines11040796)
Supplement: Supplementary file 1 [file vaccines-11-00796-s001.zip › vaccines-2303643-supplementary.pdf]

Impact of COVID-19 vaccination on short-term perceived change in physical performance among elite athletes: an international survey

Supplementary material

S1. Survey in English

Vaccination and performance in high level athletes survey

Dear Athlete,

The University of Liège and the French-speaking Olympic Network for Research in Sports Medicine (ReFORM) invite you to participate in an online survey concerning vaccination and performance in high-level athletes. You have been invited to participate because you meet the criteria for the level of sports performance we are looking for.

Your participation is entirely voluntary and anonymous.

The data collected will not identify you and will be used for research purposes only.

This data will allow us to better understand the possible links between COVID-19 vaccination and perceived sports performance in high performance athletes.

This study has been evaluated by the Ethical Committee of the University Hospital of Liège, which has given its approval for its conduct.

Thank you for your participation!

By clicking on "Next" you agree to participate in this study.

How old are you? *

Please enter your age in the list below ...

What is your nationality? *

Please choose ...

What sport do you mainly practice? *

Please choose ...

How many hours of sport do you do, on average, per week (training + competition)? *

Please choose among following answers ...

Have you received at least one dose of COVID-19 vaccine? *

- ◯ yes
- ◯ no

When did you receive your first dose of COVID-19 vaccine? *

- ◯ January 2021
- ◯ February 2021
- ◯ March 2021
- ◯ April 2021
- ◯ May 2021
- ◯ June 2021
- ◯ July 2021
- ◯ August 2021
- ◯ September 2021
- ◯ October 2021
- ◯ November 2021
- ◯ December 2021
- ◯ I don't know

With which vaccine? *

- ◯ Johnson & Johnson
- ◯ Pfizer/BioNTech
- ◯ Moderna
- ◯ AstraZeneca
- ◯ Spoutnik V
- ◯ I don't know
- ◯ Other ______

What level of side effects did you experience from the first dose of the vaccine? *

None ______ Very high

How long did side effects, if any, from the first dose last? *

- ◯ No side effects
- ◯ Less than one day
- ◯ Between 1 and 3 days
- ◯ Between 4 and 7 days
- ◯ Between 8 and 30 days
- ◯ More than 30 days

Did the side effects of the first dose impact your training? *

Yes, I trained less ______ Yes, I trained even more

When did you receive the second dose of the COVID-19 vaccine? *

- ◯ I did not receive a second dose
- ◯ January 2021
- ◯ February 2021
- ◯ March 2021
- ◯ April 2021
- ◯ May 2021
- ◯ June 2021
- ◯ July 2021
- ◯ August 2021
- ◯ September 2021
- ◯ October 2021
- ◯ November 2021
- ◯ December 2021
- ◯ I don't know

With which vaccine did you get the second dose? *

- ◯ Johnson & Johnson
- ◯ Pfizer/BioNTech
- ◯ Moderna
- ◯ AstraZeneca
- ◯ Spoutnik V
- ◯ I don't know
- ◯ Other ______

What level of side effects did you experience from the second dose of the vaccine? *

None ______ Very high

How long did side effects, if any, from the second dose last? *

- ◯ No side effects
- ◯ Less than one day
- ◯ Between 1 and 3 days
- ◯ Between 4 and 7 days
- ◯ Between 8 and 30 days
- ◯ More than 30 days

Did the side effects of the second dose impact your training? *

Yes, I trained less [slider] Yes, I trained even more

Have you been tested positive for COVID-19? *

- ◯ yes
- ◯ no

If yes, when have you been tested positive for COVID-19? (multiple answers allowed) *

- ☐ I don't know
- ☐ Before year 2021
- ☐ January 2021
- ☐ February 2021
- ☐ March 2021
- ☐ April 2021
- ☐ May 2021
- ☐ June 2021
- ☐ July 2021
- ☐ August 2021
- ☐ September 2021
- ☐ October 2021
- ☐ November 2021
- ☐ December 2021

Do you think that COVID-19 may have impacted your sports performance? *

Yes, strong deterioration [slider] Yes, strong improvement

Since your vaccination, how satisfied are you with your sports performance? *

Not satisfied at all [slider] Very satisfied

Since your vaccination, have you been injured? *

- ◯ yes
- ◯ no

After your vaccination, did you feel a deterioration or improvement in your sports performance? *

A strong deterioration [slider] A strong improvement

When was your last international competition? *

- ◯ Before the year 2021
- ◯ January 2021
- ◯ February 2021
- ◯ March 2021
- ◯ April 2021
- ◯ May 2021
- ◯ June 2021
- ◯ July 2021
- ◯ August 2021
- ◯ September 2021
- ◯ October 2021
- ◯ November 2021
- ◯ December 2021

How satisfied were you with your performance in this last international competition? *

Not satisfied at all [slider] Very satisfied

When was your biggest international objective in 2021? *

- ◯ January 2021
- ◯ February 2021
- ◯ March 2021
- ◯ April 2021
- ◯ May 2021
- ◯ June 2021
- ◯ July 2021
- ◯ August 2021
- ◯ September 2021
- ◯ October 2021
- ◯ November 2021
- ◯ December 2021

How satisfied were you with your performance in this biggest international objective? *

Not satisfied at all [slider] Very satisfied

Did you feel pressure (societal, professional, medical, ...) to get vaccinated? *

Not at all [slider] A lot

If you felt pressure, was it social pressure (family, friends, social networks, media, ...)? *

Not at all [slider] A lot

If you felt pressure, was it professional pressure (team doctor, federations, sports colleagues, travel restrictions, participation in certain competitions, etc.)? *

Not at all [slider] A lot

Do you feel that you had a free choice to get vaccinated? *

Totally [slider] Not at all

Do you feel that your full vaccination against COVID-19 has impacted your sports performance? *

Yes, a very negative impact [slider] Yes, a very positive impact

How long did this impact on your performance last? *

- ◯ No impact
- ◯ Less than one day
- ◯ Between 1 and 3 days
- ◯ Between 4 and 7 days
- ◯ Between 8 and 30 days
- ◯ More than 30 days

Do you think that vaccination against COVID-19 may have impacted the sports performance of other high-level athletes? *

Not at all [slider] Most probably

Do you have any additional comments?

Could you please tell us briefly why? *

Do you have any additional comments?

The survey is finished. Thank you very much for your participation.
This window can be closed.

S2. Survey in French

Vaccination et performance chez les athlètes de haut niveau

Chèr·e sportif·ve,

L'Université de Liège et le Réseau Francophone Olympique de la Recherche en Médecine du sport (ReFORM) vous invitent à participer à une enquête en ligne concernant la vaccination et la performance chez les athlètes de haut niveau. Vous avez été sollicité·e car vous répondez aux critères de niveau de performance sportive que nous recherchons.

Votre participation est entièrement volontaire et anonyme.

Les données collectées ne permettront pas de vous identifier et seront uniquement utilisées à des fins de recherche. Ces données nous permettront de mieux comprendre les liens éventuels entre la vaccination contre la COVID-19 et la perception des performances sportives chez les athlètes de haut niveau.

Cette étude a été évaluée par le Comité Ethique Hospitalo-Facultaire de Liège qui a donné son accord à sa réalisation.

Nous vous remercions pour votre participation !

En cliquant sur "suivant" vous consentez à participer à cette étude.

Quel âge avez-vous ? *

Veuillez indiquer dans la liste ci-dessous, votre âge...

Quelle est votre nationalité ? *

Veuillez choisir ...

Quel sport pratiquez-vous principalement ? *

Veuillez choisir parmis les réponses ci-dessous ...

Combien d'heures de sport pratiquez-vous en moyenne par semaine (entrainement + compétition)? *

Veuillez choisir parmis les réponses ci-dessous ...

Avez-vous reçu au moins une dose de vaccin contre la COVID 19 ? *

- oui
- non

Quand avez-vous reçu la première dose de vaccin contre la COVID 19 ? *

- ◯ Janvier 2021
- ◯ Février 2021
- ◯ Mars 2021
- ◯ Avril 2021
- ◯ Mai 2021
- ◯ Juin 2021
- ◯ Juillet 2021
- ◯ Aout 2021
- ◯ Septembre 2021
- ◯ Octobre 2021
- ◯ Novembre 2021
- ◯ Décembre 2021
- ◯ Je ne sais pas

Avec quel vaccin ? *

- ◯ Johnson & Johnson
- ◯ Pfizer/BioNTech
- ◯ Moderna
- ◯ AstraZeneca
- ◯ Spoutnik V
- ◯ Je ne sais pas
- ◯ Autre ____________

Quel est le niveau d'effets secondaires que vous avez ressenti suite à la première dose du vaccin? *

Aucun effet secondaire ———— Effets secondaires très importants

Combien de temps ont duré les éventuels effets secondaires de la première dose de vaccination? *

- ◯ Aucun effet secondaire
- ◯ Moins d'un jour
- ◯ De 1 à 3 jours
- ◯ De 4 à 7 jours
- ◯ De 8 à 30 jours
- ◯ Plus de 30 jours

Les effets secondaires de la première dose de vaccination ont-ils impactés votre entrainement? *

Oui, je me suis moins entrainé(e) ———— Oui, je me suis encore plus entrainé(e)

Quand avez-vous reçu la seconde dose du vaccin contre la COVID 19 ? *

- ◯ Je n'ai pas reçu de deuxième dose
- ◯ Janvier 2021
- ◯ Février 2021
- ◯ Mars 2021
- ◯ Avril 2021
- ◯ Mai 2021
- ◯ Juin 2021
- ◯ Juillet 2021
- ◯ Aout 2021
- ◯ Septembre 2021
- ◯ Octobre 2021
- ◯ Novembre 2021
- ◯ Décembre 2021
- ◯ Je ne sais pas

Avec quel vaccin avez-vous été vacciné(e) la seconde dose ? *

- ◯ Johnson & Johnson
- ◯ Pfizer/BioNTech
- ◯ Moderna
- ◯ AstraZeneca
- ◯ Spoutnik V
- ◯ Je ne sais pas
- ◯ Autre ____________

Quel est le niveau d'effets secondaires que vous avez ressenti suite à la seconde dose du vaccin? *

Aucun effet secondaire ———— Effets secondaires très importants

Combien de temps ont duré les éventuels effets secondaires de la seconde dose de vaccination? *

- ◯ Aucun effet secondaire
- ◯ Moins d'un jour
- ◯ De 1 à 3 jours
- ◯ De 4 à 7 jours
- ◯ De 8 à 30 jours
- ◯ Plus de 30 jours

Les effets secondaires de la seconde dose de vaccination ont-ils impactés votre entrainement? *

Oui, je me suis moins entrainé(e) [ ] Oui, je me suis encore plus entrainé(e)

Avez-vous été testé(e) positif(ve) à la COVID ? *

- ◯ oui
- ◯ non

Si oui, quand avez-vous été testé(e) positif(ve) à la COVID ?
(plusieurs réponses possibles) *

- ☐ Je ne sais pas
- ☐ Avant l'année 2021
- ☐ Janvier 2021
- ☐ Février 2021
- ☐ Mars 2021
- ☐ Avril 2021
- ☐ Mai 2021
- ☐ Juin 2021
- ☐ Juillet 2021
- ☐ Aout 2021
- ☐ Septembre 2021
- ☐ Octobre 2021
- ☐ Novembre 2021
- ☐ Décembre 2021

Pensez-vous que la COVID ait pu impacter votre performance sportive ? *

Oui, forte détérioration [ ] oui, forte amélioration

Depuis votre vaccination, quel est votre niveau de satisfaction quant à votre performance sportive? *

Pas du tout satisfait.e [ ] Très satisfait.e

Depuis votre vaccination, avez-vous été blessé(e) ? *

- ◯ oui
- ◯ non

Après votre vaccination, avez-vous senti une détérioration ou une amélioration de vos performances sportives? *

Une forte déterioration [ ] Une forte amélioration

De quand date votre dernière compétition internationale ? *

- ◯ Avant 2021
- ◯ Janvier 2021
- ◯ Février 2021
- ◯ Mars 2021
- ◯ Avril 2021
- ◯ Mai 2021
- ◯ Juin 2021
- ◯ Juillet 2021
- ◯ Août 2021
- ◯ Septembre 2021
- ◯ Octobre 2021
- ◯ Novembre 2021
- ◯ Décembre 2021

Quel est votre niveau de satisfaction quant à votre performance sportive lors de cette dernière compétition internationale? *

Pas du tout satisfait.e [ ] Très satisfait.e

Quand s'est déroulé votre plus grand objectif international en 2021 ? *

- ◯ Janvier 2021
- ◯ Février 2021
- ◯ Mars 2021
- ◯ Avril 2021
- ◯ Mai 2021
- ◯ Juin 2021
- ◯ Juillet 2021
- ◯ Août 2021
- ◯ Septembre 2021
- ◯ Octobre 2021
- ◯ Novembre 2021
- ◯ Décembre 2021

Quel est votre niveau de satisfaction quant à votre performance sportive lors de votre plus grand objectif international? *

Pas du tout satisfait.e [ ] Très satisfait.e

Avez-vous ressenti une pression (sociétale, professionnelle, médicale, ...) pour vous faire vacciner? *

Pas du tout [ ] énormément

Si vous avez ressenti une pression, était-ce une pression sociale (famille, amis, réseaux sociaux, médias, ...)? *

Pas du tout [ ] énormément

Si vous avez ressenti une pression, était-ce une pression professionnelle (médecin de l'équipe, fédérations, collègues sportifs, obligation pour voyager, obligation pour participer à certaines compétitions, ...)? *

Pas du tout [ ] énormément

Avez-vous le sentiment que vous avez eu un libre choix pour vous faire vacciner ? *

Totalement [ ] Pas du tout

Estimez-vous que votre vaccination complète contre la COVID-19 a impacté vos performances sportives? *

Oui, un impact très négatif ______ Oui, un impact très positif

Combien de temps a duré cet impact sur vos performances ? *

- ◯ Aucun impact
- ◯ De 1 à 3 jours
- ◯ De 8 à 30 jours
- ◯ Moins d'un jour
- ◯ De 4 à 7 jours
- ◯ Plus de 30 jours

Pensez-vous que la vaccination contre la COVID-19 ait pu impacter la performance sportive d'autres athlètes de haut niveau? *

Pas du tout ______ très probablement

Avez-vous un commentaire supplémentaire à faire ?

Pouvez-vous nous dire brièvement pourquoi ? *

Avez-vous un commentaire supplémentaire à faire ?

Le sondage est terminé. Merci beaucoup pour votre participation.

La fenêtre peut être fermée.

Pouvez-vous nous dire brièvement pourquoi ? *

Table S1. *Univariate analysis of the factors associated with the perceived negative impact on physical performance during more than 8 days after full COVID-19 vaccination*

**Characteristics**	**No negative or negative impact < 8days (n=263)**	**Negative impact > 8days (n=43)**	**P-value**
Age (years)			
< 18	46 (17.5%)	2 (4.7%)	**0.02**
18-25	137 (52.1%)	20 (46.5%)	
>25	80 (30.4%)	21 (48.8%)	
Sport			
Collective	83 (31.6%)	3 (7.0%)	**0.004**
Individual	180 (68.4%)	40 (93.0%)	
Sport by week (hours)			
Between 5 and 10	16 (6.1%)	0 (0.0%)	0.25
Between 11 and 20	132 (50.2%)	19 (44.2%)	
Between 21 and 35	105 (39.9%)	22 (51.2%)	
Between 36 and 50	10 (3.8%)	2 (4.6%)	
Type of vaccine			
AstraZeneca	6 (2.3%)	1 (2.3%)	0.97
Johnson & Johnson	25 (9.5%)	3 (7.0%)	
Moderna	13 (4.9%)	2 (4.7%)	
Pfizer/BioNTech	219 (83.3%)	37 (86.0%)	
**First dose of vaccine (n=306)**			
Level of side effects (VAS)	10 (0-35)	40 (15-80)	**< 0.0001**
Duration of side effects			
No side effects	85 (32.3%)	3 (6.9%)	**< 0.0001**
Less than 1 day	57 (21.7%)	4 (9.3%)	
Between 1 and 3 days	115 (43.7%)	18 (41.9%)	
More than 3 days	6 (2.3%)	18 (41.9%)	
Impact on training (VAS)	50 (30-50)	20 (0-50)	**<0.0001**
**Second dose of vaccine (n=294)**			
Level of side effects (VAS)	10 (0-40)	70 (50-90)	**< 0.0001**
Duration of side effects			
No side effects	101 (40.2%)	3 (7.0%)	**< 0.0001**
Less than 1 day	38 (15.1%)	4 (9.3%)	
Between 1 and 3 days	95 (37.8%)	9 (20.9%)	
More than 3 days	17 (6.9%)	27 (62.8%)	
Impact on training (VAS)	48 (26-50)	10 (0-32.5)	**<0.0001**
Injury since vaccination			
Yes	90 (34.2%)	16 (37.2%)	0.70
No	173 (65.8%)	27 (62.8%)	
Pressure to get vaccinated	30 (0-70)	80 (60-100)	**<0.0001**
Positive for COVID-19 before first dose			
Yes	50 (19%)	8 (18.6%)	0.95
No	213 (81%)	35 (81.4%)	
